# Nutritional Composition and Functional Potential of Groundnut (*Arachis hypogaea* L.) Landraces Indigenous to the Gamo Zone of Ethiopia

**DOI:** 10.1002/fsn3.71356

**Published:** 2025-12-29

**Authors:** Yonas Syraji, Jeyaramraja Papanasam Rajamanickam, Asfaw Abadi, Dawit Albene, Fikru Alemu

**Affiliations:** ^1^ Department of Biology, College of Natural Sciences Arba Minch University Arba Minch Ethiopia; ^2^ PG and Research Department of Botany PSG College of Arts & Science, Civil Aerodrome Post Coimbatore India; ^3^ Department of Economics, College of Business and Economics Arba Minch University Arba Minch Ethiopia; ^4^ Ministry of Innovation and Technology Addis Ababa Ethiopia

**Keywords:** antioxidant activity, elemental analysis, groundnut landraces, proximate composition, seed characterization

## Abstract

Groundnuts ( *Arachis hypogaea* L.) are an important oilseed crop grown in more than 100 countries worldwide. In this study, various groundnut landraces in the Gamo Zone of Southern Ethiopia were examined for their seed characterization, proximate composition, antioxidant activity, phenol content, and elemental content. The aim of this study was to evaluate the nutritional and biochemical diversity among these landraces to identify those with superior nutritional and functional properties. The mean ± standard deviation was used to report mean values. Key characteristics such as the number of seeds per pod, 100‐pod weight, 100‐seed weight, individual seed weight, and shelling percentage showed significant differences (*p* < 0.05) between landraces. Interestingly, Kucha Alpha's Wonde and Sete landraces continuously displayed higher values than their Boreda and Kucha. Proximate analysis showed that the landraces differed significantly in terms of moisture, ash, carbohydrate, protein, fat, and amino acid content. In addition to ash and carbohydrate contents varying among groups, Wonde Kucha Alpha's moisture content was substantially different from that of Wonde and Sete of Kucha. Significant variations in carbohydrates were observed in the Sete Boreda landrace, but not in the Harar landrace. Different nutritional profiles were indicated by the significant differences in the protein and amino acid contents of Kucha Alpha landraces compared to Harar of Boreda. Analysis of fat content revealed notable differences between Kucha Alpha and Wonde Boreda landraces. Significant antioxidant activity was found in methanolic extracts, with Kucha Alpha Wonde having the highest total phenolic content (153.38 ± 0.05). Furthermore, elemental analysis with XRF spectroscopy showed that the landraces' mineral composition has different elemental composition. These results provide important information for breeding and nutritional enhancement by highlighting the genetic and biochemical diversity among Ethiopian groundnut landraces.

## Graphical abstract

Significant morphological, biochemical, and nutritional variability was observed among groundnut landraces, highlighting rich genetic diversity and functional potential. Certain accessions, particularly from Boreda, showed superior protein, fat, and phenolic content with strong antioxidant activity. These findings suggest their value for breeding, nutritional enhancement, and functional food development, promoting sustainable use of Ethiopia's indigenous groundnut genetic resources.

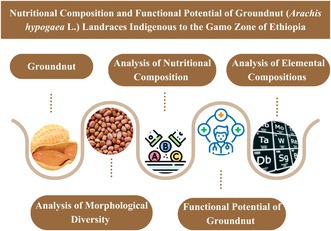

## 1 Introduction

An annual legume, groundnuts ( *Arachis hypogaea* L.) are also known as peanuts, earthnuts, monkey nuts, and goobers. It is grown in more than 100 countries across six continents and is one of the most significant oilseed crops in the world, ranking as the fourth most important oilseed crop and the thirteenth most important food crop (Ephrem 2015). In addition to being high in vitamin E, niacin, riboflavin, thiamine, folacin, calcium, phosphorus, magnesium, zinc, iron, and potassium, groundnut kernels have 40%–50% fat, 20%–50% protein, and 10%–20% carbohydrates (Ephrem 2015).

For many small businesses, groundnuts provide substantial cash income. Producers and the nation's foreign exchange profits from exports (Tarekegn et al. 2007). The demand for groundnuts in global markets has been increasing gradually over time. Due to the snack food markets in North America and the EU, as well as in nations where groundnuts are a staple in foods like Mediterranean, Indian, and Asian, the demand for groundnuts seems to be stable going forward (Rios and Jaffee 2008). In many developing nations, groundnuts are essential to the rural economy and the livelihoods of impoverished farmers (Daniel 2009). Challenges in groundnut production include pests, diseases, climate change, market fluctuations, and sustainability concerns. Efforts to develop improved varieties resistant to diseases and pests are ongoing to ensure the sustainability and profitability of the global groundnut industry (Alade et al. 2021).

In Ethiopia, groundnuts are one of the five oilseed crops that are grown extensively (Sori 2021). Compared to other regions of the country, the eastern Hararghe zone of the Oromia region holds a leading position in terms of production and supply for both domestic and export markets (Gezahagn 2013). The areas in Gamo Zone that have the potential to produce are Boreda, Kucha, and Kucha Alpha.

The production of groundnuts is essential to the local economy and food security in Gamo Zone, especially in the districts of Kucha, Kucha Alpha, and Boreda. Due to the favorable agroclimatic conditions in these regions, smallholder farmers grow groundnuts alongside other crops (Tekle et al. 2022). There are several groundnut landraces in those districts: Sete, Wende, and Hareri in Boreda, and Sete and Wende in Kucha and Kucha Alpha. Farmers find the Sete and Wende groundnut landraces to be valuable due to their unique characteristics and features.

The high yield potential and strong disease resistance of the Sete groundnut landraces are well‐known. Farmers prefer this landrace because of its productivity and ability to adapt to different growing environments. Farmers can use Sete groundnuts because they are usually medium‐sized and have a good flavor and texture. On the other hand, Wende groundnut landraces are highly valued for their potential yield and resistance to disease. Compared to other Sete landraces, Wende groundnuts are distinguished by their larger seeds. When compared to other varieties, Hareri groundnut landraces are renowned for having larger seeds. One significant factor that affects the groundnut's market value is the size of the seeds. Prior to conducting a hybridization experiment to produce improved varieties with heterosis, one trait that is crucial in the selection of parent plants is the size of the seed.

Yet, no research has been done to analyze the nutritional makeup of groundnut landraces in those regions. Our aim in this study is to thoroughly assess the total phenol content, antioxidant activities, proximate composition, and seed characterization. To further describe the elemental makeup of groundnut landraces, we will perform XRF analysis. By clarifying the nutritional makeup of groundnut landraces, this study aims to offer important information about their seed characterization, elemental composition, antioxidant activities, phenol content, and proximate composition (such as ash, moisture, CHO, protein, amino acid, and fat content). Additionally, it offers information for crop improvement and product development.

## 2 Material and Methods

### 2.1 Description of Sampling Site

**FIGURE 1:**
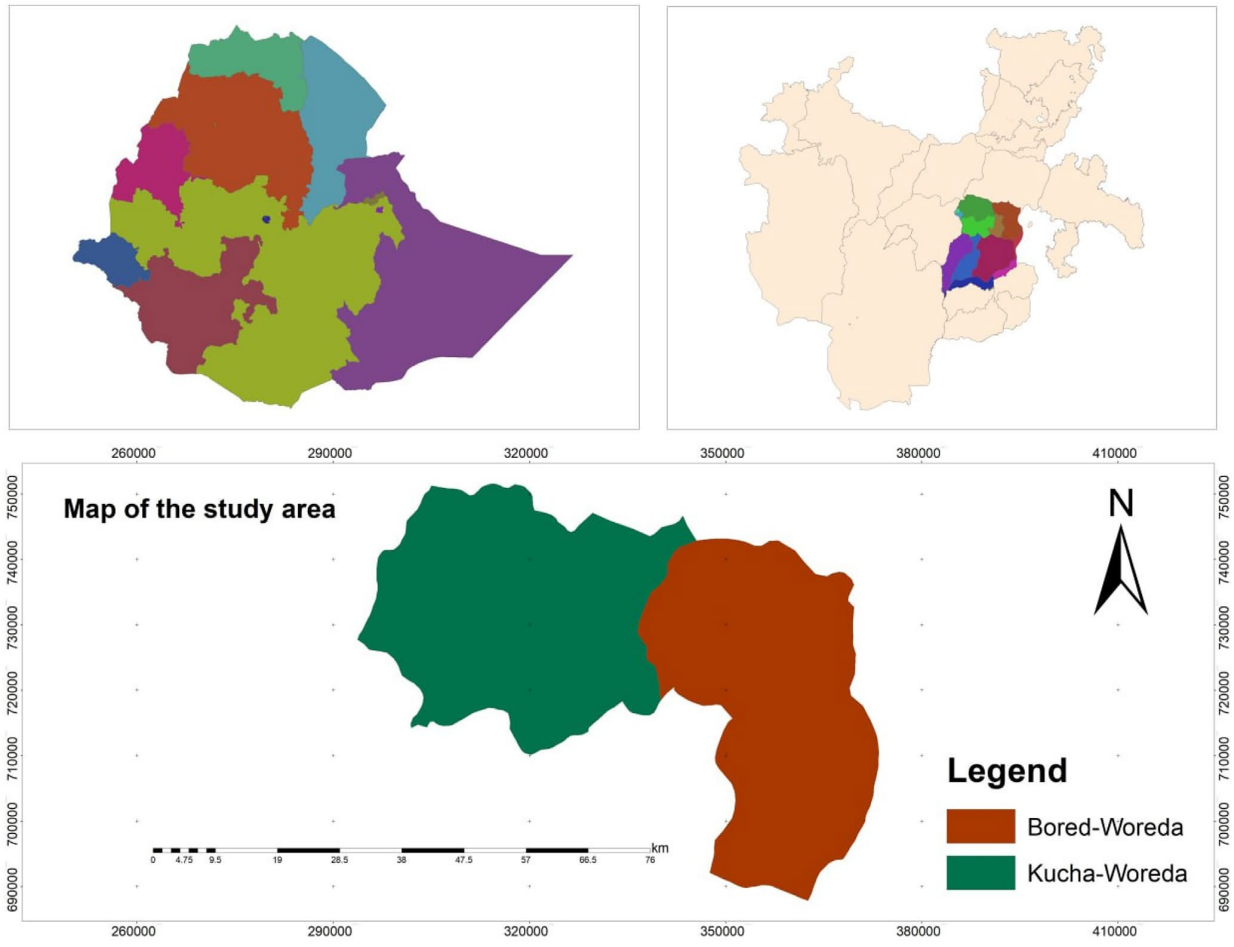
Map showing the study area.

Groundnuts were collected from three districts in the Gamo Zone of the Southern Nations, Nationalities, and Peoples' Region (SNNPR), Ethiopia: Kucha, Kucha Alpha, and Boreda as shown in Figure 1. These areas are characterized by a bimodal rainfall pattern, with the main rainy seasons occurring from March to April and June to August, and altitudes ranging from 900 to 2400 m above sea level. The mean annual temperature across the sites is approximately 17.5°C–22.5°C. Soils are predominantly volcanic in origin, with reddish‐brown to dark‐brown textures.

### 2.2 Study Design

A purposive study design strategy was applied during the collection of groundnut landraces samples. Three areas were selected from Gamo zone. The areas were selected based on their potential of groundnut production.

### 2.3 Sample Collection

The groundnut samples were collected randomly from the areas of Kucha, Kucha Alpha, and Boreda. The samples were transported to Arba Minch University, Abaya Campus, Biology laboratory, and kept under room temperature until analysis begins.

### 2.4 Seed Characterization

In order to describe the pods and seeds of these groundnut landraces, the current study examined parameters like individual seed weight, number of seeds per pod, 100 pod weight, 100 seed weight, and shelling percentage (Jeyaramraja and Fantahun 2014).

### 2.5 Proximate Composition

#### 2.5.1 Moisture Content Determination

According to AOAC (2003) method 925, the dry air oven method was used to determine the moisture content of samples of groundnut landraces. 10.2 g of each sample were dried in a hot air oven at 105°C for 24 h and the drying was proceeding until constant weight obtained. Moisture content was then calculated as:

(1) $$\begin{array}{c} \text{Moisture content}\;\left(\%\right)\\ =\frac{\text{Weight of fresh sample}-\text{Weight of}\;\mathrm{dry}\;\text{sample}}{\text{Weight of fresh sample}}\times 100 \end{array}$$

#### 2.5.2 Determination of Ash Content

AOAC (1995) method No. 923‐09 was used to determine the sample's ash content (AOAC 1995). A clean porcelain dish was ignited for 3 h at 550°C in a muffle furnace and weighed as (W1) after being cooled in a desiccator. After that, 5 g of the sample flour was added to the porcelain dish and weighed as (W2). These samples were carbonized on a hot plate after being dried at 120°C for an hour. After the dish and its contents were moved to a muffle furnace, they were ignited at 550°C until the ashing process was finished. (W3) was the weight of the residue. The percentages of total ash on a dry matter basis were as follows:

(2) $$\begin{array}{c} \mathrm{Ash}\;\text{content}\;\left(\%\right)\\ =\frac{\text{Weight of}\;a\;\text{sample}-\text{Weight of empty crucible}}{\text{Weight of crucible with}\;\mathrm{ash}-\text{Weight of empty crucible}}\times 100 \end{array}$$

#### 2.5.3 Estimation of Total Carbohydrate

The methods described in the literature (Yonas and Jeyaramraja 2022; Sadasivam and Manickam 2008; Yonas et al. 2025) were used to measure total carbohydrates. 100 mg of groundnut powdered seeds were hydrolyzed with 5 mL of 2.5 N HCl in a boiling water bath for 3 h, cooled, neutralized with sodium carbonate, and diluted to 100 mL in order to estimate the amount of carbohydrates. Following centrifugation, 0.5 and 1 mL aliquots of the supernatant were taken. In order to bring the total volume in all tubes (including samples) up to 1 mL with distilled water, standards were made using 0–1 mL of a 100 μg/mL glucose working standard. After adding 4 mL of ice‐cold anthrone reagent to each tube, the mixture was heated for 8 min in a boiling water bath, cooled, and its absorbance at 630 nm was measured. A standard curve was plotted, and total carbohydrates were expressed as mg glucose per 100 mg sample (percent equivalent of glucose).

#### 2.5.4 Estimation of Protein

Total proteins were measured using the methods described in the literature (Yonas and Jeyaramraja 2022; Sadasivam and Manickam 2008; Yonas et al. 2025). By grinding 500 mg of groundnut seeds in 5 mL of phosphate buffer (with pH 7) and centrifuging the homogenate to extract the supernatant, protein estimation was carried out. Test tubes containing 0.1 and 0.2 mL of sample extract and bovine serum albumin (BSA) standards (0–1 mL, 200 μg/mL) were filled with distilled water to bring the volumes down to 1 mL. After 5 mL of alkaline copper solution and 10 min of incubation, 0.5 mL of Folin–Ciocalteu reagent was added to each tube. Absorbance was measured at 660 nm following a half‐hour dark incubation period at room temperature. Protein content was computed as mg protein/100 mg sample (% equivalent of BSA) using a standard curve.

#### 2.5.5 Estimation of Total Free Amino Acids

The procedures described in the literature (Yonas and Jeyaramraja 2022; Sadasivam and Manickam 2008; Yonas et al. 2025) were followed in the assay of total free amino acids. 5 mL of hot 80% ethanol were used to extract 500 mg of groundnut seed powder in order to estimate the total free amino acids. The assay was performed using the centrifuged supernatant. 1 mL of ninhydrin solution was added to 0.1 mL of extract (or leucine standards, 0–1 mL, 100 μg/mL), and the volume was adjusted to 2 mL using distilled water. Following 20 min of heating in a boiling water bath, the tubes were cooled and combined with 5 mL of diluent (equal parts water and n‐propanol). The absorbance at 570 nm was measured after 15 min in comparison to a reagent blank. Leucine equivalents as a percentage of total free amino acids were used.

#### 2.5.6 Estimation of Crude Fat Contents

Using 150 mL of diethyl ether, 2 g of the sample was extracted in the Soxhlet extractor for 8 h. The solvent was heated in a steam bath to evaporate it. The extracted fat‐containing flask was dried for 1 h at 100°C in a drying oven, and the mass was calculated using AOAC (2000) method No. 30‐10 (AOAC 2000). In the end, it was taken out and allowed to cool in a desiccator. The formula below was used to determine the crude fat content.

(3) $$\begin{array}{c} \mathrm{Fat}\;\text{content}\;\left(\%\right)\\ =\frac{\text{Weight of flask}+\mathrm{Fat}-\text{Weight of empty flask}}{\text{Weight of sample taken}}\times 100 \end{array}$$

### 2.6 X‐Ray Fluorescence Spectroscopy (XRF) Analysis

A 100 g sample was used for XRF analyses. An ORTECR multichannel analyzer, a semiconductor detector Si/Li (beryllium window thickness = 0.25 mm, beryllium window diameter = 5 mm), and a radionuclide source of radiation 238Pu (A = 370 MBq, E = 12–22 keV, T = 86.4 years) manufactured by Amersham in the form of a planar disk source comprised the XRF measuring system. A non‐coaxial geometrical arrangement of source, sample, and detector was used for all measurements, and the acquisition time was 2000 s (Annalakshmi et al. 2012).

### 2.7 2, 2‐Diphenyl‐1picrylhydrazyl (DPPH) Radical Scavenging Assay

DPPH radical scavenging by extracts from groundnut landraces was calculated using a previously published methodology (Irshad et al. 2012). 2 mL of extracts in varying concentrations (200–800 mg/L) were combined with 2 mL of DPPH solution in methanol (0.004%, 0.102 mM). The extracts were used to correct the baseline at 515 nm and were replaced with methanol for the blank solution. 20 min were spent letting the tubes stand at room temperature. The plant extract's capacity to reduce DPPH radicals served as the basis for determining its antiradical activity. Using a linear curve of the ascorbic acid standard, the scavenging effect of the groundnut landraces extracts was assessed. Ascorbic acid was used as a standard in the range of 25–100 mg/L.

### 2.8 Determination of Total Phenol by Folin‐Ciocalteau Reagent Method

With some modifications, the Folin‐Ciocalteau reagent method was used to determine the total phenol content. 1 mg of each groundnut landraces crude extract was dissolved in 1 mL of methanol. Folin‐Ciocalteau reagent (10 mL) was added to 90 mL of water to create a 10% solution. Next, 2.5 g of Na_2_CO_3_ was dissolved in 50 mL of water to create 5% Na_2_CO_3_. 1.5 mL of 10% Folin‐Ciocalteau reagent was added to 200 μL of each crude extract in a test tube. After that, each test tube was left in a dark area for 5 min. Lastly, the solutions were thoroughly mixed by hand after 1.5 mL of 5% Na_2_CO_3_was added. Once more, all of the test tubes were left in the dark for 2 h. A UV spectrophotometer was used to measure the absorbance of each solution at a fixed wavelength of 750 nm. A linear gallic acid standard curve was utilized to ascertain the phenol content of the plant extracts. Gallic acid was used as a standard in the range of 2.5–100 mg/L (Kaur and Kapoor 2002).

### 2.9 Data Analysis

All quantitative data were obtained as the mean of three determinations ± standard error. Statistical analyses were performed using SPSS version 25 for Windows. One‐way Analysis of Variance (ANOVA) was used to assess differences among landraces, followed by Tukey's HSD post‐hoc test to determine significant differences between mean values at *p* < 0.05.

## 3 Result and Discussions

### 3.1 Seed Characterization

#### 3.1.1 Number of Seeds per Pod

The results for number of seeds per pod of groundnut landraces are presented in Table 1. A significant variation (*p* < 0.05) was observed in the number of seeds per pod among the studied landraces. Specifically, the Wonde and Sete landraces of Kucha Alpha exhibited significantly higher values compared to their counter parts from Boreda. However, no statistically significant difference was found between these landraces of Hareri. This variation could be attributed to genetic differences among the landraces or environmental factors influencing seed development. These findings are consistent with previous research on groundnut genetic diversity. Yami and Abtew (2025) reported substantial genetic variability among groundnut genotypes for yield‐related traits, including seed number and kernel weight, indicating the potential for trait improvement through selective breeding (Harika et al. 2025). Similarly, Harika et al. (2025) observed high levels of phenotypic and genotypic variation in pod and kernel traits, which supports the superior performance observed in certain landraces in the current study (Harika et al. 2025). Additionally, Rajanna et al. (2024) identified significant genetic divergence among groundnut germplasm lines, particularly in pod weight and seed characteristics, further corroborating the distinct genetic potential of the studied landraces (Rajanna et al. 2024).

**TABLE 1:** Seed and pod characteristics of groundnut in Gamo landraces.

Parameters	Kucha landraces		Kucha Alpha landraces		Boreda landraces		
	Wende	Sete	Wende	Sete	Wende	Sete	Hareri
Number of seeds per pod	2.00 ± 0.05	1.07 ± 0.02	2.30 ± 0.10	2.2 ± 0.10	2.00 ± 0.04	1.8 ± 0.01	2.10 ± 0.01
100 pods weight	97.33 ± 0.10	147.18 ± 0.01	110.77 ± 0.01	115.34 ± 0.01	93.15 ± 0.01	101.40 ± 0.20	126.03 ± 0.00
100 seed weight	37.91 ± 0.63	54.33 ± 0.30	45.02 ± 0.01	61.52 ± 0.01	44.97 ± 0.02	44.35 ± 0.30	59.71 ± 0.10
Individual seed weight	0.38 ± 0.02	0.54 ± 0.04	0.45 ± 0.04	0.62 ± 0.01	0.45 ± 0.30	0.44 ± 0.02	0.60 ± 0.00
Shelling percent	85.60 ± 0.10	72.23 ± 0.20	78.47 ± 0.20	63.92 ± 0.02	86.88 ± 0.10	75.80 ± 0.20	75.12 ± 0.02

#### 3.1.2 100‐Pod Weight

The results for 100‐Pod Weight of groundnut landraces are presented in Table 1. The 100‐pod weight, a key agronomic trait associated with yield, showed no significant difference between the Kucha and Kucha Alpha landraces. However, a statistically significant difference (*p* < 0.05) was observed between the Sete landraces of Kucha and Boreda, suggesting varietal differences that may influence pod development and filling. These findings are consistent with studies that report significant genotypic variations in 100‐pod weight among groundnut varieties. Akpalu et al. (2012) investigated five Bambara groundnut landraces: Nav 4, Nav Red, Black Eye, Mottled Cream, and Burkina and found that pod yield was significantly affected by plant spacing, with wider spacing producing higher pod yields (Akpalu et al. 2012). This implies that both genotype and agronomic practices can influence pod development. Similarly, Donkor et al. (2022) reported highly significant genetic variation (*p* < 0.001) in traits closely related to pod characteristics, such as grain yield, reinforcing the role of genetic diversity in shaping pod weight and yield potential among landraces (Donkor et al. 2022).

#### 3.1.3 100‐Seed Weight

The results for 100‐Seed Weight of groundnut landraces are presented in Table 1. A significant difference (*p* < 0.05) was observed between Sete Kucha Alpha and the Wonde and Sete landraces of Boreda, indicating variability in seed size among these genotypes. In contrast, no significant differences were found between the Kucha and Boreda landraces, suggesting potential similarities in seed size. These findings align with previous studies that report significant genotypic variations in 100‐seed weight among groundnut landraces. For instance, a study by Damfami et al. (2024) evaluated 17 Bambara groundnut landraces and found that 100‐seed weight exhibited high broad‐sense heritability (75.20%) and a genetic advance as a percentage of the population mean (92.53%), indicating substantial genetic variability for this trait (Damfami et al. 2024). Similarly, Donkor et al. (2022) assessed 25 Bambara groundnut accessions and reported highly significant differences (*p* < 0.001) among almost all traits studied, including 100‐seed weight, with high heritability values, suggesting that this trait is predominantly governed by additive gene action and can be effectively improved through selection (Damfami et al. 2024).

#### 3.1.4 Individual Seed Weight

The results for individual seed weight of groundnut landraces are presented in Table 1. Significant differences (*p* < 0.05) were observed between the Wonde and Sete landraces of Boreda and the Sete landrace of Kucha Alpha. However, no significant difference was found between the Kucha and Boreda landraces, suggesting comparable seed development patterns. These findings align with previous research highlighting genetic variability in seed weight among groundnut landraces. For instance, a study by Syraji Yonas et al. (2025) investigated various Ethiopian groundnut landraces and varieties, revealing significant variation (*p* < 0.05) in individual seed weight (ISW) (Yonas et al. 2025). Notably, varieties from Haramaya exhibited higher mean ISW compared to those from the Gamo zone and Werer, indicating that genetic factors significantly influence seed weight among different landraces. Similarly, research by Khaliqi et al. (2021) assessed multiple Bambara groundnut genotypes and found significant differences in hundred seed weight, a trait closely related to individual seed weight (Khaliqi et al. 2021). The study reported that genotype G5LR1P3 had the highest hundred seed weight (121.26 g), while G3SR1P1 had the lowest (56.13 g), underscoring the substantial genetic variability in seed weight traits among landraces.

#### 3.1.5 Shelling Percentage

The results for shelling percentage of groundnut landraces are presented in Table 1. Significant differences (*p* < 0.05) were observed between the Wonde and Sete landraces of Kucha Alpha and the Wonde landrace of Boreda. However, no significant difference was found between the Kucha Alpha and Sete landraces of Boreda. These findings align with previous research highlighting genetic variability in shelling percentage among groundnut landraces. For instance, Akpalu et al. (2012) evaluated five Bambara groundnut landraces Nav 4, Nav Red, Black Eye, Mottled Cream, and Burkina and found that Mottled Cream recorded the highest shelling percentage of 70.6%, significantly surpassing the other landraces (Akpalu et al. 2012). This suggests that genetic factors play a crucial role in determining shelling efficiency among different landraces. Similarly, a study by Magagula et al. (2019) investigated the effects of plant density and planting patterns on groundnut yield components in Eswatini. The study reported that shelling percentage varied with plant density, with the highest shelling percentage of 59.67% observed at an intermediate plant density of 44,444 plants per hectare.

### 3.2 Proximate Analysis

**FIGURE 2:**
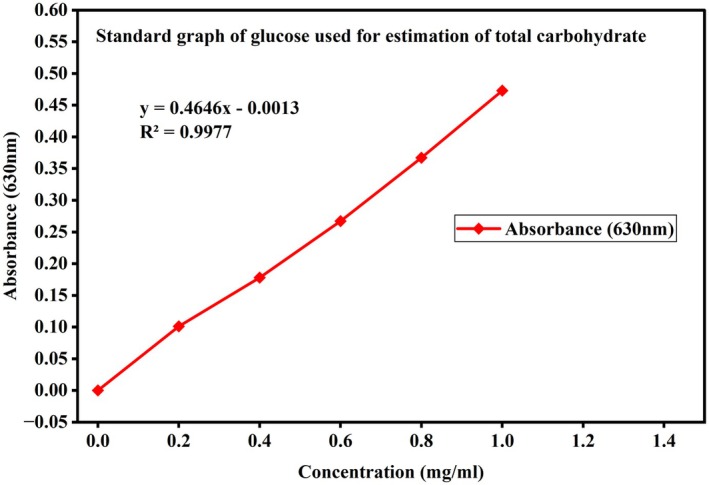
The standard graph used for the estimation of carbohydrate.

**FIGURE 3:**
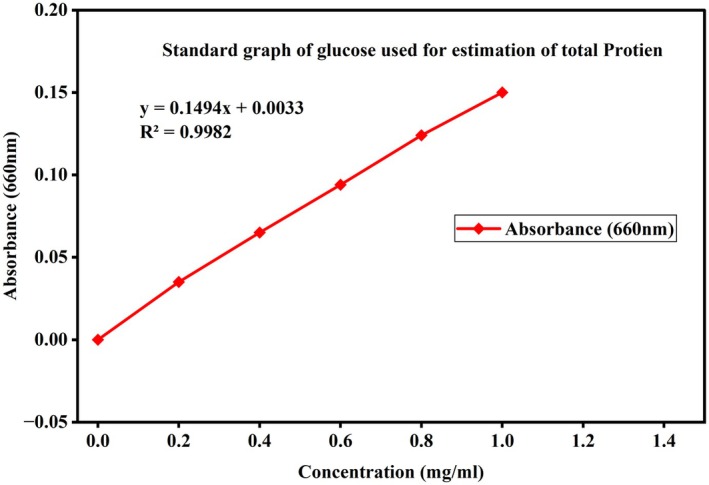
The standard graph used for the estimation of total proteins.

**FIGURE 4:**
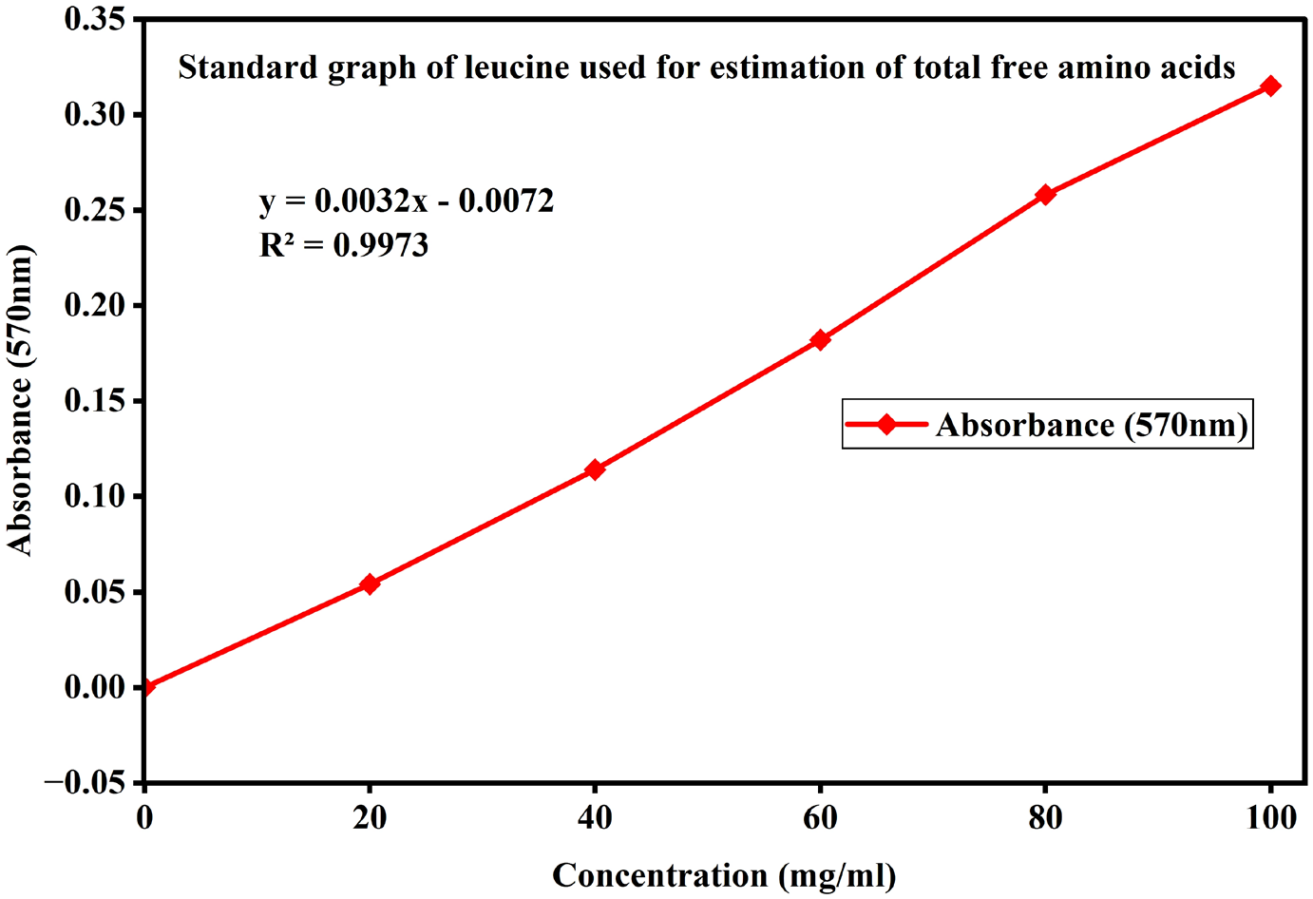
The standard graph used for the estimation of total free amino acids.

Proximate analysis, namely moisture, ash, carbohydrates, protein, amino acid, and fat were measured in Gamo zone groundnut landraces. The standard graphs used for the calculation of carbohydrate, protein, and free amino acid in the groundnut samples are presented in Figures 2, 3, 4. Data on moisture, ash, carbohydrates, protein, amino acid, and fat in Gamo zone groundnut landraces are shown in Table 2.

**TABLE 2:** Proximate analysis of groundnut landraces in Gamo zone.

Parameter	Kucha landraces		Kucha Alpha landraces		Boreda landraces		
	Wende	Sete	Wende	Sete	Wende	Sete	Hareri
Moisture (%)	6.02 ± 0.20	5.96 ± 0.20	6.90 ± 0.10	6.20 ± 0.20	6.20 ± 0.20	6.22 ± 0.10	6.10 ± 1.0
Ash (%)	2.07 ± 0.02	2.18 ± 0.10	2.89 ± 0.10	2.68 ± 0.10	2.86 ± 0.10	2.71 ± 0.10	1.92 ± 0.02
CHO (mg/mL)	3233.11 ± 0.10	3544.84 ± 0.04	2845.76 ± 0.10	3856.54 ± 0.30	4065.04 ± 0.04	2053.39 ± 0.30	2375.30 ± 0.30
Protein (mg/mL)	4.88 ± 0.00	5.61 ± 0.01	4.85 ± 0.10	5.28 ± 0.10	6.56 ± 0.10	8.26 ± 0.10	8.26 ± 0.10
Amino acid (mg/mL)	205.56 ± 0.10	201.49 ± 0.10	333.33 ± 0.10	287.27 ± 0.20	281.21 ± 0.20	250.00 ± 1.00	254.71 ± 0.02
Fat (%)	48.30 ± 0.20	57.60 ± 0.30	41.00 ± 1.00	49.80 ± 0.05	61.60 ± 0.30	52.00 ± 1.00	58.60 ± 0.10

In the present study, the moisture content of Wonde Kucha alpha has shown statistically significant difference (*p* < 0.05) with Wonde and Sete Kucha landraces. At the same time, Sete Kucha alpha has shown significant difference with Harare at (*p* < 0.05), but no significant difference was observed between Wonde Kucha alpha Harare at (*p* = 0.55). Each landrace's moisture content falls below the 10% safe storage limit, which lowers the possibility of aflatoxin contamination and microbial growth while being stored. Our results are in line with those of Sahdev et al. (2017), who used forced convection drying experiments to record comparable moisture levels in Indian groundnuts. Under controlled conditions, they found that groundnut kernels' moisture content could be effectively lowered to less than 7%, enhancing storability and reducing postharvest losses.

In this study, it was observed that the ash content of Wonde Kucha, Boreda, and Kucha alpha landraces has shown significant difference at (*p* < 0.05) but no significant difference between Sete Kucha, Kucha alpha, and Boreda. In other cases, the ash content of groundnut landraces of Harare showed significant difference (*p* < 0.05) with Wonde and Sete of both Kucha alpha and Boreda landraces, respectively. Ash content is a crucial measure of nutritional value since it represents the seeds' overall mineral concentration. Similar studies were also found by Hama‐Ba et al. (2022), who found that groundnut varieties grown in Burkina Faso with varying soil types had ash contents ranging from 1.8% to 2.3%. Their research brought to light how soil composition, especially clay‐rich soils, affects the buildup of minerals in seeds.

The CHO of Sete Boreda showed significant difference with both Sete Kucha and Kucha alpha at (*p* < 0.05) but it was not significantly difference with groundnut landrace of Harare at (*p* = 0.5537). The results shown here align with those of Ameha and Leta (2020), who noticed that raw groundnut samples from Ethiopia's East Hararghe Zone had carbohydrate contents ranging from 40.2% to 41.71%. Groundnut seeds' higher carbohydrate content is advantageous because it offers an intense energy source. Therefore, for breeding programs intended to increase the nutritional value of groundnuts, landraces with a higher carbohydrate content are preferred.

The other important proximate parameter was determination of protein content and the results showed that the protein content of Wonde and Sete of Kucha alpha groundnut landrace was significantly different from Harar landraces at (*p* < 0.05) and also Kucha alpha Wonde showed significant difference with Boreda Wonde and Sete landrace at (*p* < 0.05). The nutritional significance of Ethiopian groundnut landraces was further highlighted by Ameha and Leta (2020), who reported protein levels ranging from 24.5% to 26.0%.

The amino acid content of both Wonde and Sete of Kucha alpha showed a significant difference (*p* < 0.05) with Kucha groundnut landraces, but the amino acid of Harare has no significant difference with all of the other groundnut landraces. Similar amino acid compositions in groundnut seeds have been reported in earlier research. Olayinka et al. (2022) discovered that groundnut proteins are abundant in leucine, lysine, and valine essential amino acids that are critical to human health.

In this study, the fat content of Wonde Boreda has shown a significant difference with both Wonde and Sete of Kucha alpha at (*p* < 0.05) but there was no significant difference observed between Wonde of Kucha and Kucha alpha respectively. The results of this study are supported by Ameha and Leta (2020), who found that Ethiopian groundnuts had fat contents ranging from 46.4% to 51.2%. Landrace variations in fat content can be explained by soil fertility, environmental variables like temperature and rainfall, and genotypic variability. While landraces with moderate fat levels might be suitable for direct consumption applications where lower oiliness is preferred, those with consistently higher fat content might be more desirable for the oil extraction industries. According to the findings, groundnuts are a great source of oil, and the high‐fat landraces that have been identified may be specifically encouraged for commercial cultivation and oil production.

### 3.3 Antiradical Activity of Plant Extracts

**FIGURE 5:**
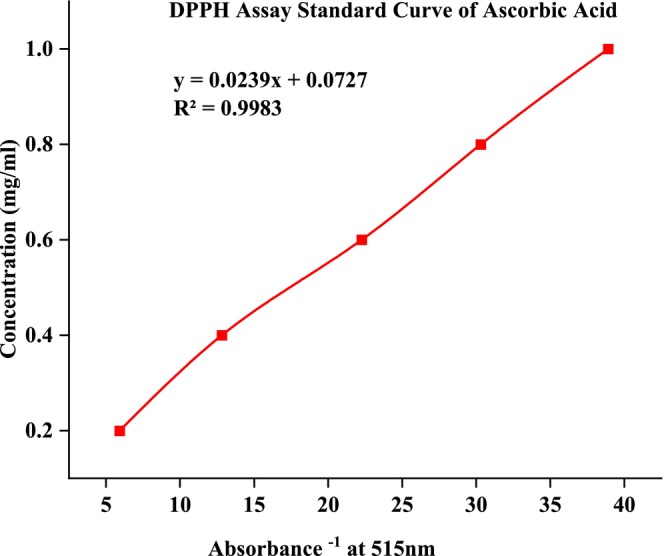
Standard curve of ascorbic acid.

By contrasting the percentage DPPH scavenging activity with a reference, ascorbic acid, the groundnut landrace extracts' DPPH radical scavenging capabilities were calculated (Figure 5). In this work, the radical‐scavenging effects were investigated using DPPH, a stable free radical with a distinctive absorption at 515 nm. Because it can bind transition metal ions and scavenge free radicals, ascorbic acid is regarded as a potent antiradical and was used as a standard.

**FIGURE 6:**
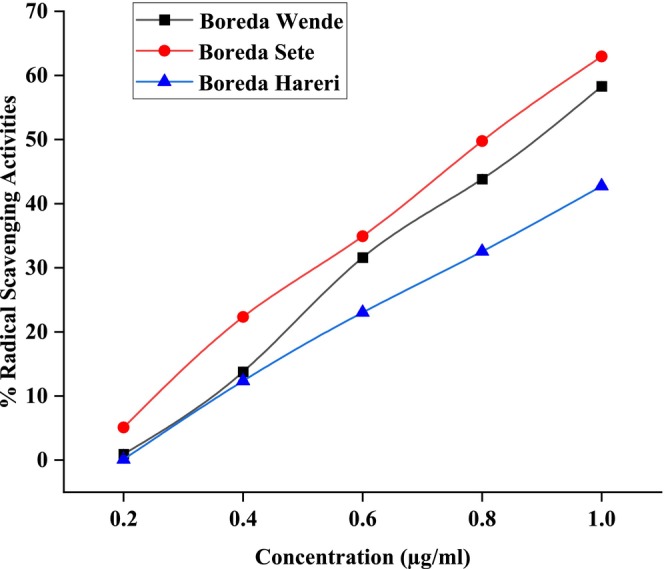
Radical scavenging activity (% RSA) of groundnut landraces of Boreda.

**FIGURE 7:**
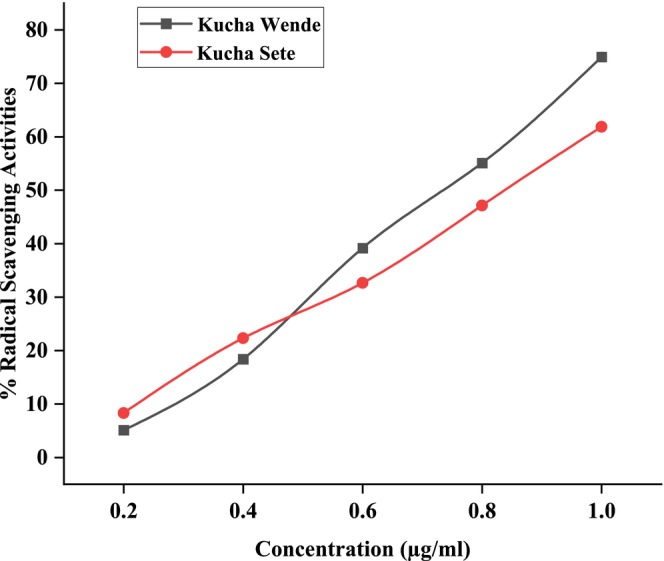
Radical scavenging activity (% RSA) of groundnut landraces of Kucha.

**FIGURE 8:**
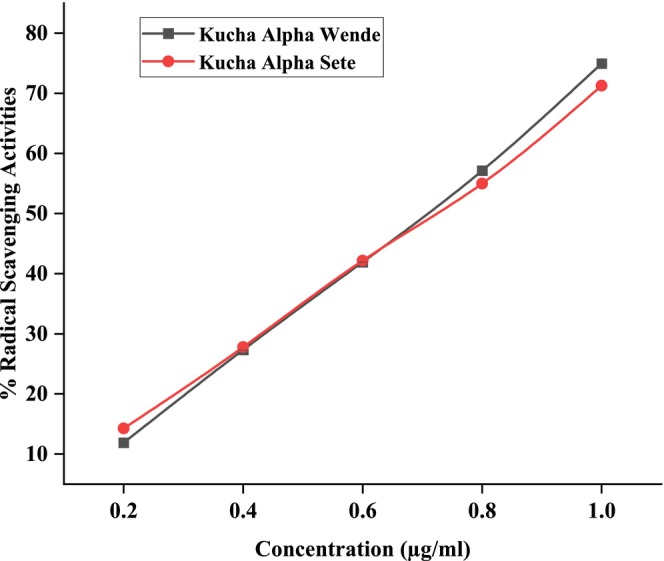
Radical scavenging activity (% RSA) of groundnut landraces of Kucha Alpha.

**FIGURE 9:**
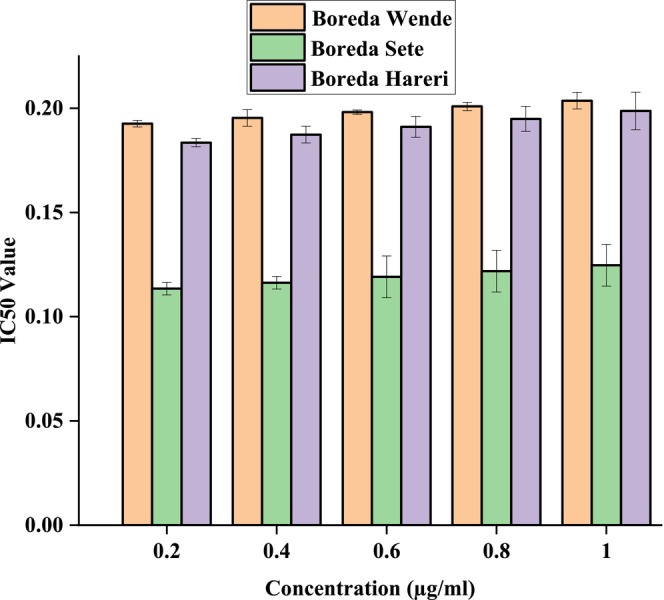
IC_50_ values of groundnut landraces of Boreda.

**FIGURE 10:**
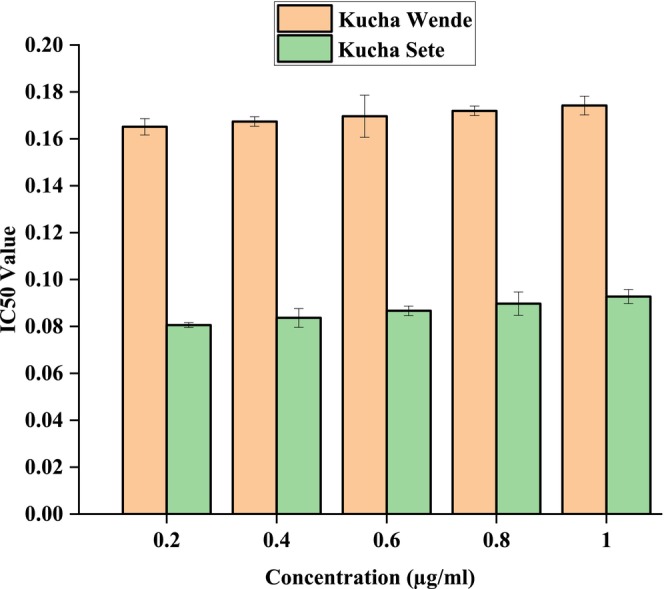
IC_50_ values of groundnut landraces of Kucha.

**FIGURE 11:**
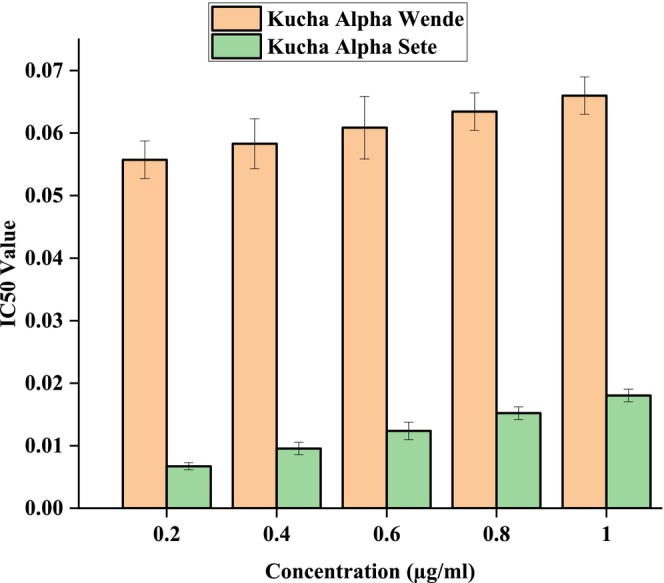
IC_50_ values of groundnut landraces of Kucha Alpha.

Figures 6, 7, 8 showed the results of DPPH free radical scavenging. The amount of radical scavenging can be assessed by the decline in absorption. The values of the radical‐scavenging activity were represented as the ratio of the absorbance of the DPPH solution without extract at 515 nm to the percentage of sample absorbance decrease. As demonstrated by the IC50 value in Figures 9, 10, 11, groundnut landraces were found to inhibit the DPPH free radical scavenging activity. This indicates that it has a significant antiradical effect on DPPH's free radical scavenging activity.

In general, among all the groundnut landraces extracts, the crude extracts obtained from groundnut landraces of Kucha Wende extracts showed the best antiradical activity on the other hand, groundnuts landraces of Kucha Alpha Sete and Boreda Sete extract shows lower antiradical activities respectively. A correlation between the total phenolic content and antioxidant activity has been found by some authors (Miliauskas et al. 2004). Strong metal chelation properties may be exhibited by antioxidant compounds. They serve as reducing agents, donate hydrogen, and quench singlet and triplet oxygen due to their redox properties (Kähkönen et al. 1999). The antioxidant capacity of the extract is determined by its antioxidant content, as well as by the interactions and structures of these molecules (Bourgou et al. 2008). It was assessed how different concentrations of ascorbic acid and groundnut landrace extracts inhibited the scavenging of DPPH. Figures 9, 10, 11 showed the plant extracts' IC50 values.

The crude extracts from Kucha Alpha Sete exhibited the highest IC50 value among all the groundnut landrace extracts, indicating the lowest antioxidant potency, as a higher IC50 reflects weaker free radical scavenging activity. In contrast, the extracts from Kucha Sete and Bored Sete showed notably lower IC50 values, suggesting stronger antioxidant activity and greater efficiency in neutralizing free radicals. These differences imply that the Kucha Sete and Bored Sete landraces possess superior antioxidant potential, which may be associated with higher concentrations of phenolic compounds or other bioactive metabolites contributing to their enhanced free radical scavenging capacity.

### 3.4 Phenol Content

**FIGURE 12:**
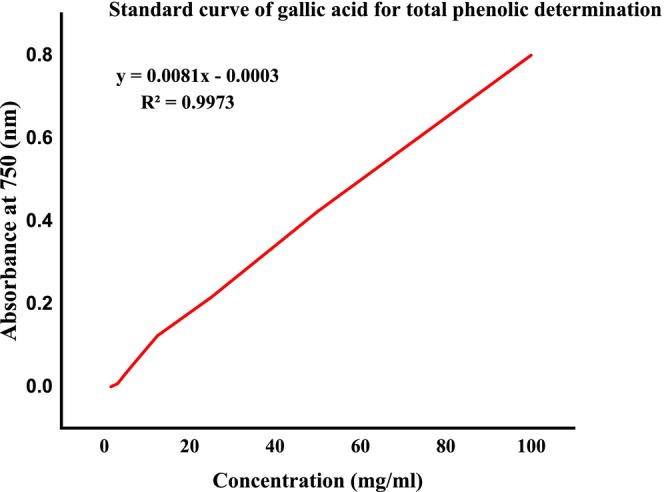
Total phenolic content for standard gallic acid; values expressed in terms of gallic acid.

The amount of phenolic compounds in groundnut landrace plant extracts was calculated using the regression equation of the gallic acid calibration curve (Figure 12) and reported in Table 3 as milligrams of gallic acid equivalent per gram of dry extract (mg GAE/g).

**TABLE 3:** Phenolic content.

Area	Landraces	Total phenolic content (μg/mL)
Kucha	Wende	78.32 ± 0.10
	Sete	90.41 ± 0.30
Kucha Alpha	Wende	153.38 ± 0.05
	Sete	97.95 ± 0.05
Boreda	Wende	85.85 ± 0.03
	Sete	80.29 ± 0.04
	Hareri	97.58 ± 0.04

The total phenolic content calculated in this study is presented in Table 3. The highest value of phenolic content was obtained in Kucha alpha Wende, which is 153.38 ± 0.05 methanol extract, followed by Kucha alpha Sete methanol extract, which is 97.95 ± 0.04, while Kucha Wende methanol extract shows lower phenolic content, which is 78.32 ± 0.10 as shown in Table 3.

Antioxidant activity is known to be directly impacted by strong chain‐breaking antioxidants like phenol (Aghraz et al. 2018; Alam et al. 2018). The nuclear structure of these phenolic compounds is arranged with functional groups (hydroxyl) for hydrogen donation to stabilize radical molecules, which contributes to antioxidant activity (Soobrattee et al. 2008).

### 3.5 XRF Analysis of Groundnut Landraces Plant Parts

In this work, XRF spectroscopy was used to determine the percentage and concentration of different elements in groundnut landraces Table 4. The elemental analysis of groundnut landraces from Kucha, Kucha Alpha, and Boreda places revealed notable variation in mineral composition. Boreda Sete landraces exhibited consistently higher concentrations of Calcium (Ca) measured 3079.4 ± 107.14 ppm, potassium (K) measured 27277.93 ± 330.2 ppm, sulfur (S) measured 2793.25 ± 321.84 ppm, and zinc (Zn) measured 43.99 ± 4.42 ppm, indicating strong nutritional potential. In contrast, Kucha and Kucha Alpha landraces, particularly Kucha Alpha Wende, showed elevated levels of iron (Fe) measured 138.66 ± 17.21 ppm and molybdenum (Mo) measured 7.91 ± 1.45 ppm. Elements such as uranium and copper were detected only in specific landraces, while zirconium and strontium levels remained relatively stable across all samples.

**TABLE 4:** Evaluation of elemental content of groundnut landraces in Gamo zone by X‐ray fluorescence spectroscopy.

Kucha land races				Kucha alpha land races				Boreda land races					
Wende		Sete		Wende		Sete		Wende		Sete		Hareri	
Elem	Con	Elem	Con (ppm)	Elem	Con (ppm)	Elem	Con (ppm)	Elem	Con (ppm)	Elem	Con (ppm)	Elem	Con (ppm)
Mo	9.77 ± 1.41	Mo	9.98 ± 1.62	Mo	7.91 ± 1.45	Mo	7.47 ± 1.46	Mo	8.23 ± 1.46	Mo	7.7 ± 1.36	Mo	6.66 + 1.36
Zr	9.27 ± 1.3	Zr	8.55 ± 1.48	Zr	9.13 ± 1.35	Zr	8.36 ± 1.34	Zr	9.56 ± 1.36	Zr	8.14 ± 1.25	Zr	8.03 ± 1.26
Sr	12.69 ± 1.04	Sr	12.09 ± 1.18	Sr	11.85 ± 1.06	Sr	10.61 ± 1.04	Sr	11.16 ± 1.05	Sr	10.26 ± 0.97	Sr	12.81 ± 1.03
U	3.64 ± 2.16	U	0	U	0	U	6.71 ± 2.37	U	0	U	0	U	3.49 ± 2.12
Rb	5.67 ± 1.11	Rb	5.74 ± 1.25	Rb	8.96 ± 1.22	Rb	6.76 ± 1.22	Rb	10.6 ± 1.28	Rb	10.15 ± 1.18	Rb	5.15 ± 1.08
Zn	37.93 ± 4.37	Zn	52.77 ± 5.5	Zn	40.89 ± 4.62	Zn	35.5 ± 4.5	Zn	44.1 ± 4.75	Zn	43.99 ± 4.42	Zn	37.52 ± 4.3
Cu	12.49 ± 6.48	Cu	0	Cu	0	Cu	0	Cu	0	Cu	13.99 ± 6.27	Cu	0
Fe	64.78 ± 14.64	Fe	121.52 ± 18.64	Fe	138.66 ± 17.21	Fe	59.4 ± 15.21	Fe	64.72 ± 15.32	Fe	19.8 ± 3.13	Fe	40.14 ± 13.79
Ca	2499.63 ± 84.79	Ca	1632.64 ± 56.44	Ca	2220.77 ± 74.02	Ca	1784.88 ± 65.1	Ca	2156.12 ± 75.56	Ca	3079.4 ± 107.14	Ca	1729.91 ± 76.93
K	17803.27 ± 245.78	K	11941.16 ± 164.76	K	17837.99 ± 221.91	K	14244.74 ± 195.08	K	19898.38 ± 235.33	K	27277.93 ± 330.2	K	19243.88 ± 251.64
S	1063.01 ± 212.85	S	863.56 ± 147.62	S	1472.53 ± 202.64	S	1142.14 ± 182.6	S	1862.83 ± 220.91	S	2793.25 ± 321.84	S	1621.97 ± 235.47

*Note:* Key Elem = Element, Con = Concentration in ppm.

The elemental analysis revealed notable variations in zinc (Zn), iron (Fe), and calcium (Ca) contents among the groundnut landraces from the Gamo Zone (Table 4). The Fe concentration ranged from 19.8 ± 3.13 ppm in Boreda Sete to 138.66 ± 17.21 ppm in Kucha Alpha Wende, while Zn content varied from 35.5 ± 4.5 ppm in Kucha Alpha Sete to 52.77 ± 5.5 ppm in Kucha Sete. Similarly, Ca content ranged between 1632.64 ± 56.44 ppm in Kucha Sete and 3079.4 ± 107.14 ppm in Boreda Sete. These results indicate that the mineral profiles differ significantly across landraces, suggesting genotypic variation and possible environmental influences on mineral uptake, as also reported in other groundnut studies (Janila et al. 2014).

From a nutritional perspective, these differences are important because Fe, Zn, and Ca are essential micronutrients for human health. Iron supports hemoglobin synthesis and oxygen transport, zinc plays a crucial role in immune response and enzymatic regulation, and calcium contributes to bone development and muscle function (Suliburska and Krejpcio 2011). When compared to human dietary standards (FAO/WHO recommended daily intakes of approximately 8–18 mg Fe, 8–11 mg Zn, and 1000 mg Ca for adults), groundnuts from the Gamo Zone, particularly Kucha Alpha Wende (for Fe) and Boreda Sete (for Ca)—could provide a meaningful portion of daily mineral requirements if consumed regularly (Anthony et al. 2020). The relatively high Fe and Ca concentrations observed in these landraces highlight their potential nutritional value and suitability for promoting dietary diversity and combating micronutrient deficiencies in local communities. Similar findings have been reported in other studies emphasizing the mineral richness and dietary significance of peanuts and related legumes (Anthony et al. 2020).

These differences may be attributed to genetic diversity among the landraces, as well as environmental and soil‐related factors. The findings highlight the potential of specific landraces for targeted nutritional and agronomic use, supporting future efforts in crop selection and improvement. These results shed light on the elemental profile of groundnut landrace extracts and highlight the possible nutritional benefits of these elements. The groundnut landraces' mineral composition showed that all of the mineral elements were present; the interaction of the trace mineral composition found in medicinal plants is crucial to comprehending how they work in the human body (Syraji et al. 2024).

## 4 Conclusions

This study revealed significant variability among the groundnut landraces in morphological, nutritional, and biochemical traits, highlighting rich genetic diversity and potential for targeted selection. The observed differences in seed characteristics, proximate composition, antioxidant activity, and elemental content underscore the influence of both genetic and environmental factors. Certain landraces, particularly those from Boreda and Kucha Alpha Wende, exhibited superior nutritional and antioxidant profiles, indicating their promise for use in breeding programs and functional food development. Overall, these landraces represent valuable genetic resources for improving groundnut quality, enhancing food security, and promoting human nutrition. Future research should focus on evaluating their field performance, adaptability, and post‐harvest stability to support sustainable utilization and conservation efforts.

## Author Contributions

**Yonas Syraji:** investigation, writing – original draft, methodology, writing – review and editing, software, formal analysis, supervision, conceptualization. **Jeyaramraja Papanasam Rajamanickam:** writing – original draft, writing – review and editing, supervision, conceptualization. **Asfaw Abadi:** formal analysis, conceptualization, and visualization. **Dawit Albene:** methodology, conceptualization, visualization, and validation. **Fikru Alemu:** formal analysis, validation, conceptualization and validation.

## Funding

The authors have nothing to report.

## Ethics Statement

The authors have nothing to report.

## Consent

The authors have nothing to report.

## Conflicts of Interest

The authors declare no conflicts of interest.

## Data Availability

The article contains all the required information.
